# Influence of Long-Term Use of American Football Helmets on Concussion Risk

**DOI:** 10.3390/brainsci14060537

**Published:** 2024-05-24

**Authors:** Yuelin Zhang, Mayuko Mitsui, Satoru Yoneyama, Shigeru Aomura

**Affiliations:** 1 Faculty of Science and Technology, Sophia University, Tokyo 102-8554, Japan; 2 College of Science and Engineering, Aoyama Gakuin University, Tokyo 150-8366, Japanyoneyama@me.aoyama.ac.jp (S.Y.); 3 College of Science and Engineering, Tokyo Metropolitan University, Tokyo 192-0397, Japan; aomura-shigeru@hotmail.co.jp

**Keywords:** American football, long-term used helmet, concussion risk, finite element method, motion analysis

## Abstract

In this study, to discuss the influence of concussion risk from the long-term use of American football helmets on collegiate teams, accident cases during the game are replicated based on game videos by simulations using whole-body numerical models and helmeted finite element human head models. The concussion risks caused by collisions were estimated using the mechanical parameters inside the skull obtained from finite element analyses. In the analyses, the different material properties of helmets identified by free-fall experiments using headform impactor-embedded helmets were used to represent brand-new and long-term-use helmets. After analyzing the five cases, it was observed that wearing a new helmet instead of a long-term-use one resulted in a reduction in the risk of concussion by 1 to 44%. More energy is attenuated by the deformation of the liners of the brand-new helmet, so the energy transferred to the head is smaller than that when wearing the long-term-use helmet. Thus, the long-term use of the helmet reduces its ability to protect the head.

## 1. Introduction

In American football, which is a full-contact sport, head injuries due to athlete collisions are frequently reported. To protect players from head injuries, the rules of the game are being reviewed, and appropriate blocking and tackling techniques are being introduced. Following 32 deaths caused by head and neck trauma in American football in 1968, the National Operating Committee on Standards for Athletic Equipment (NOCCAE) was launched in November 1969 to establish standards for athletic equipment [1]. Subsequently, the incidence of severe injuries decreased significantly from 4.25% to 0.68% when the first helmet safety standard was established in 1973 [2]. However, while protection against fatal collisions involving skull fractures has been successful, no clear evidence suggests that helmets can reduce the occurrence of concussions. Significantly high shock attenuation is required for athletic equipment because concussions develop even with a relatively weak impact. In addition, in the performance test conducted on helmets to check whether they meet the criteria, only three to five impacts are applied to each part of the helmet, and the influence of repeated impacts on the shock attenuation properties has not been clarified [3]. Although the number of severe injury cases has decreased due to the improvement in the protection abilities of helmets, as mentioned above, the concussion incidence in collegiate athletes still remains higher than that in professional athletes. Helmet usage status also appears to be a crucial factor, in addition to the technical inexperience of colligate athletes. In professional leagues such as the NFL, helmets are frequently changed, whereas in student sports such as club activities, the same helmet is used over a long period of three or four years because of the associated costs and difficulties with its replacement. Helmets that have been used for several years exhibit scratches and dents on the outer shells and cracks on the liner; therefore, there is concern that head protection performance deteriorates owing to changes in their shape and material properties. Therefore, clarifying the change in the head protection performance of helmets due to deterioration is important because their performance is one of the significant factors in head injury occurrence.

It has been reported that traumatic brain injury can be evaluated quantitatively through responses to mechanical parameters inside the skull [4,5,6,7]. Injury risk curves have been proposed to evaluate the incidence of concussions calculated from mechanical parameters. The mechanical parameters are measured using acceleration sensors embedded in a helmet during games or obtained from the reproduction analysis of collisions using dummies and collision analysis using finite element (FE) human head models. To quantitatively evaluate concussion on the field side, authors have proposed a system to estimate concussion risk using simulations based on a game video of a collision [8,9]. In this system, the motion of the players is replicated using whole-body numerical models, and the mechanical parameters are calculated using collision analysis with helmeted finite element human head models. In this study, the assessment of long-term helmet efficacy against head impact was undertaken. Through an analysis of five concussive impact cases, utilizing the proposed risk estimation system, the associated risks of concussion for each case were evaluated within the framework of wearing either a brand-new or a long-term-use helmet.

## 2. Materials and Methods

### 2.1. Injury Estimation System

The videos used in this study were recorded during practice sessions and games of the American football team at Nihon University. Videos recorded during games often have constantly changing camera angles and the frequent use of zoom, depending on the game situation. Additionally, there are occasions where videos are only recorded from one direction, based on the circumstances. Since this study assumes the analysis of such videos, traditional methods such as the Direct Linear Transformation (DLT) method or stereo method, which measure the three-dimensional position coordinates of feature points using multiple cameras, cannot be used. This is because these methods require fixed camera angles, uniform shooting conditions, and synchronization between cameras during camera calibration. Therefore, motion analysis in MADYMO is necessary to obtain the three-dimensional posture and velocity of the head just before the collision.

**Figure 1:**
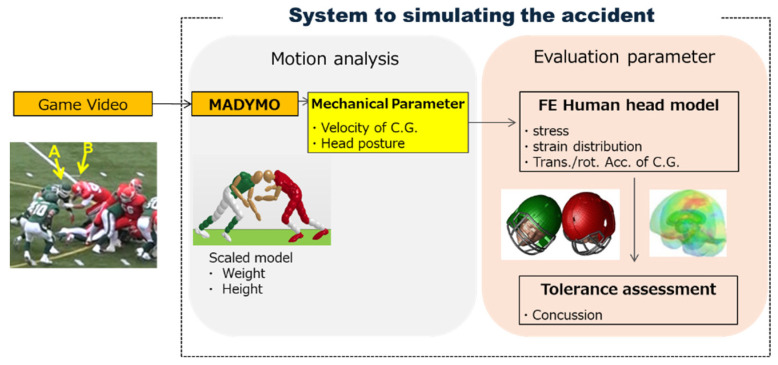
Injury estimation system based on game video using motion and collision analyses [8].

An outline of the method proposed in this study is depicted in Figure 1. The relative velocities of athletes were derived from pre- and post-collision images using Image J (Version 1.51i). Subsequently, these velocities served as input data for the scaled mathematical dynamic model of each athlete, taking into account their respective body height and weight (MADYMO ver. 7.5, TASS). Human MADYMO models were validated using postmortem human tests [10]. Due to the unknown contact characteristics between protective equipment, in the dynamic analysis, protective equipment such as a helmet was not worn, and the built-in contact characteristics of the MADYMO model were used. The position of the center of gravity (C.G.) of the head just prior to the collision (posture of head) and the translational and angular velocities of the C.G. of the heads were calculated accordingly. These parameters were then utilized as initial conditions for the collision analysis in the helmeted head finite element (FE) model (LS-DYNA ver.8.0, Ansys). The intracranial dynamic parameters, such as head accelerations, von Mises stress (vMS), and strain in the brain, were determined. The concussion risk was estimated using a predictor combining the translational acceleration of head (head motion) and vMS of the brain (brain deformation) (Equation (1)) [11].

(1) $$p=\frac{1}{1+e^{\left(5.8-0.0038a-0.76vMS\right)}}$$

where *a* is the maximum value of the translated acceleration and *vMS* is the maximum value of the von Mises stress.

### 2.2. Body Mathematical and FE Models

MADYMO is used as a global standard for dummy injury prediction and the optimum design of occupant restraint equipment. In this study, the players were represented by scaling the height and weight of the adult male model embedded in the software.

The FE head model is composed of the facial skin, scalp, skull, cerebrospinal fluid, cerebrum, cerebellum, corpus callosum, ventricle, and brain stem. The three-layered structure of the skull, comprising the outer table, diploe, and inner table, was also replicated. In this model, the falx and tentorium consist of shell elements, while the others consist of hexagonal elements. The FE model comprises 89,226 nodes and 74,462 elements, with a total mass of 4.2 kg. The material properties of each part of the model are detailed in Table 1. Elastic properties were assigned to the scalp, skull, falx, and tentorium, while viscoelastic properties were assigned to the CSF, cerebrum, brain stem, corpus callosum, and ventricle. The model was verified by comparing the accelerations, pressure, stress, and deformation of the brain to those obtained from cadaver experiments conducted in previous studies [8].

**Figure 2:**
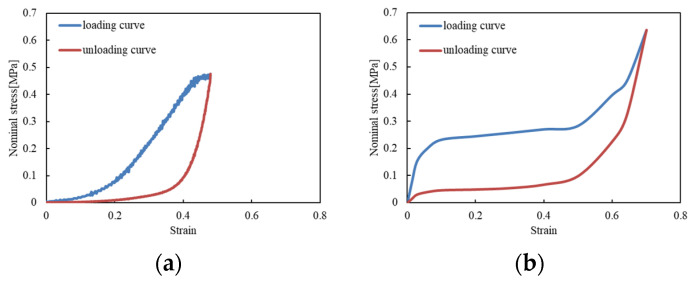
Stress–strain curves of the liner. (**a**) Brand new; (**b**) long-term use.

The FE helmet model was constructed based on the geometry of the revolution speed (Riddell), which was the same type used by the injured players during the game. The outer shell was meshed by shell elements with a constant thickness of 4.5 mm and modeled using the law of elasticity. The liner was meshed with hexagonal solid elements obtained from the extrusion of the outer shell, with a total thickness of 35 mm, and the characteristics were derived from experimental drop tests [8]. In drop testing, the time history of acceleration during impact is obtained using a triaxial accelerometer attached to the center of gravity of the head impactor. This time history of acceleration can be integrated once to obtain the time history of velocity and twice to obtain the time history of displacement. By dividing the displacement by the thickness of the liner, strain can be obtained. Furthermore, multiplying the mass of the head impactor (4.5 kg) by the time history of acceleration allows the impact force during impact to be obtained, and dividing by the contact area yields stress. The stress–strain diagram obtained by this method is applied to reproduce the analysis of drop testing, and the stress–strain diagram is fine-tuned until the acceleration of the center of gravity of the head impactor obtained in the analysis matches the measured acceleration. Figure 2 shows the obtained stress–strain curves of the liner. In this study, due to the higher resistance over time of the faceguard and shell compared to the liner, brand-new and long-term-use helmets were represented by assigning the same material properties to the faceguard and shell, while allocating different material characteristics to the liner. The long-term-use helmet had been used for one and a half years by athletes belonging to the Nihon University American football team.

## 3. Results

### 3.1. Motion Analysis of Head-to-Head Impact Accident Cases

**Figure 3:**
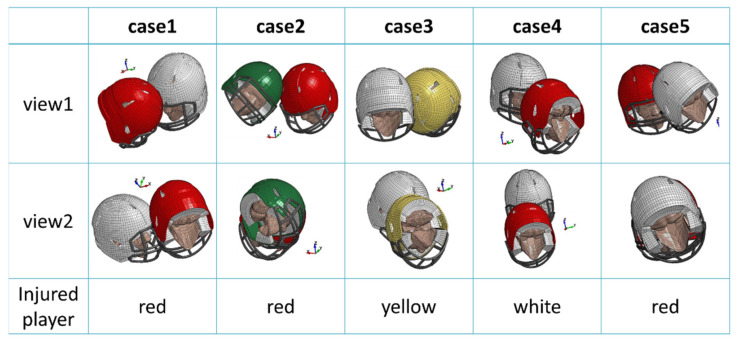
Initial positions of collision analyses of head-to-head collision accident cases.

In this study, five accident cases were collected from the Nihon University American football team over the last three years and analyzed using an injury estimation system. As an example, the details of Case 1 are presented in this study. In this case, the injured player was a defensive back player who struck the left side of his head. The athletic trainer promptly removed the player from the game due to a suspected concussion. Due to concerns about an emergent injury, the player was taken to the emergency room, where an appropriate trauma evaluation based on the guidelines of the American Academy of Neurology 2013 was performed, including a head CT, and no remarkable symptoms were observed. The player was then diagnosed with a concussion by a medical team doctor. The velocities immediately before the collision and the postures of the heads in the two directions obtained from the motion analyses of the five cases are shown in Table 2 and Figure 3, respectively.

### 3.2. Mechanical Parameter Obtained inside the Skull and Injury Estimation

**Figure 4:**
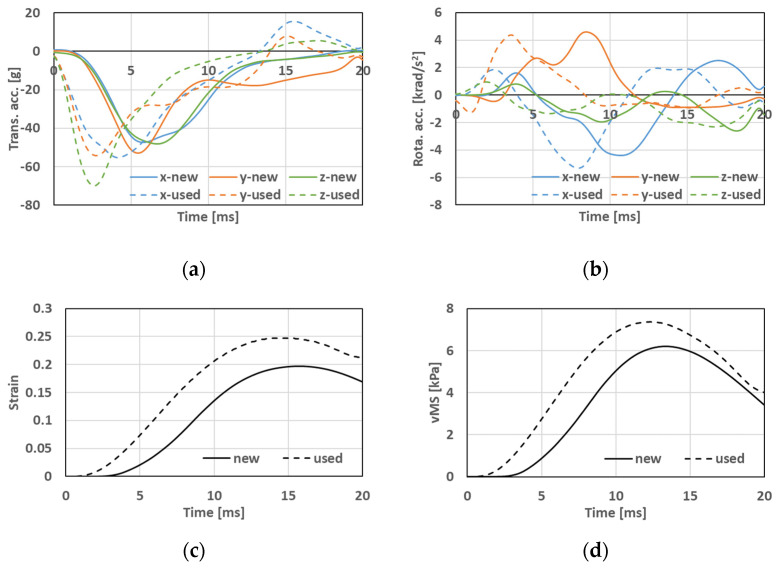
Calculated time histories of mechanical parameters inside the skull of Case 1, along with (**a**) translational accelerations, (**b**) rotational accelerations, (**c**) strain, and (**d**) vMS.

The mechanical parameters inside the skull were calculated when wearing the brand-new helmet or long-term-use helmet used in the collision analyses. As an example, the time histories of the obtained accelerations of the center of mass of the head model, the maximum principal strain (strain), and the von Mises stress (vMS) inside the skull in Case 1 are shown in Figure 4. From this figure, it can be observed that wearing a long-term-use helmet resulted in higher mechanical loads generated inside the cranium compared to wearing a brand-new helmet.

**Figure 5:**
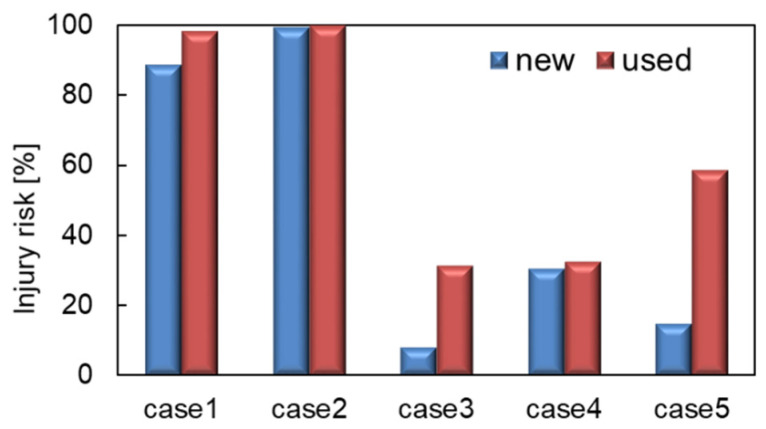
Estimated concussion risk of five cases.

The estimated concussion risks, found using Equation (1) for all cases, are shown in Figure 5. In each case, wearing a new helmet rather than one used long-term resulted in a decrease in the risk of concussion by 10%, 1%, 23%, and 2%, respectively. The results show that the risks are lower when wearing a brand-new helmet than when wearing long-term-use helmets.

## 4. Discussion

The translational acceleration obtained in this study was 88 ± 32 g, and the rotational acceleration was 3528 ± 1737 rad/s^2^. Viano et al. (2007) reported that the translational acceleration for causing concussion was 94 ± 28 g and the rotational acceleration for causing concussion was 6432 ± 1813 rad/s^2^ by reconstructing the collision motion using a Hybrid III dummy based on the game video [12]. The accelerations obtained in this study were found to be within a reasonable range. Therefore, it can be said that the impact examined in this study, demonstrating the effects of long-term helmet use, is at a level sufficient to cause concussion.

**Figure 6:**
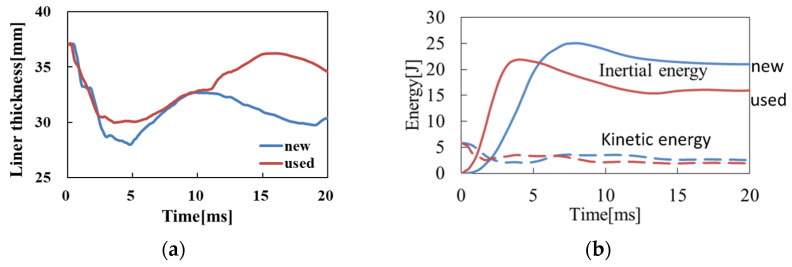
Time histories of the thickness of liners and energy of the helmet models worn by the injured player in Case 1 obtained from collision analyses: (**a**) time histories of liner thickness; (**b**) time histories of inertial and kinetic energy of the liners of helmet models.

**Figure 7:**
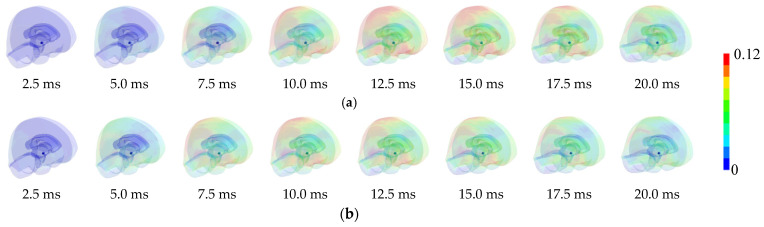
Strain distributions inside the skull of the injured player when wearing the brand-new or long-term-use helmets. (**a**) Results when wearing long-term-use helmets; (**b**) Results when wearing a new helmet.

In Case 1, the translational/rotational accelerations generated at the center of mass of the head were smaller when wearing the new helmet than when wearing long-term-use helmets. To demonstrate the behavior of the head, the time histories of the thickness of the helmets at the collision location and the time histories of the inertial and kinetic energies of the liners of the injured player are shown in Figure 6. The results show that the deformation of the liner of the brand-new helmet was larger, and the duration of deformation was longer than that of the long-term-use helmet. Therefore, the inertial energy of the new helmet increased because of the deformation of the liner, although the kinetic energies of the helmets scarcely changed; thus, greater impact energy was attenuated. In the two analyses, the collision velocities and the initial posture of the heads were the same; therefore, the impact energies applied to the head models were also the same when wearing the brand-new helmet or after long-term use. More energy is attenuated by the deformation of the liners of the brand-new helmet, so the energy transferred to the head is considered to be smaller than that when wearing the long-term-use helmet, resulting in the translational/rotational accelerations occurring at the center of gravity of the head becoming smaller. Furthermore, the time histories of the strain (the scale of the deformation) distributions inside the skull are shown in Figure 7. The maximum value of the strain decreases when a brand-new helmet is worn, and the area showing a higher value of strain (0.12 is the tolerance value of concussion, 50% probability) also becomes smaller. In other words, the concussion risk was reduced in both the evaluation predictor based on acceleration, indicating the movement of the head, and the evaluation predictor indicating the deformation of the brain. Based on these, it can be concluded that the hardness of the deformation of the liner in the long-term-use helmet reduced the shock attenuation ability, leading to a high concussion risk. This indicates that the protective performance of helmets on the head is lowered by long-term use.

The results of the reduced risk in Cases 3, 4, and 5 also have the same tendency as in Case 1; however, in Case 2, no reduction in the estimated concussion risk was observed, even when wearing a brand-new helmet. This is because the concussion risk in this case reached 100%. However, the same tendency as in the other cases was obtained because the values of the mechanical parameters were smaller for the brand-new helmet than those for the long-term-use helmet. The estimated concussion risk reached 100% because the value of the estimation predictor was high, even when wearing the new one. In general, the symptoms of concussion disappeared within one week; however, in this case, the symptoms continued for two weeks. In this case, the injury was more severe. Therefore, the brand-new helmet can decrease the energy transmitted to the head through impact attenuation. However, even if brand-new helmets can protect the head from impact, there is a limit to their protection performance.

In the five cases analyzed in this study, the results showed changes in the risk of concussion between long-term-use helmets and new helmets. However, due to the small sample size, statistical significance could not be demonstrated. Future analyses should include collisions that do not result in concussions and use statistical methods to establish significance.

## 5. Conclusions

To show the influence of the long-term use of American football helmets on concussion risk, five accident cases of head-to-head impact during a game were replicated using the brain injury estimation system. The results show that a new helmet has better protection capability against head impact than a long-term-use helmet. Thus, from the perspective of head protection, it is recommended to wear a new helmet rather than one that has been in use for one and a half years.

## Figures and Tables

**Table 1:** Material properties of the FE human head model [8].

	Scalp	Facial Bone	Outer/Inner Table	Diploe	CSF/ Ventricle	Cerebrum	Brain Stem	Corpus Callosum	Falx/ Tentorium	Cerebellum
Density ρ [kg/m^3^]	1000	1213	1456	850	1040	1040	1040	1040	1130	1040
Young’s Modulus E [MPa]	16.7	5000	5000	2320	-	-	-	-	31.5	-
Bulk Modulus K [MPa]	-	-	-	-	2190	2190	2190	2190	-	2190
Short Time Shear Modulus G_0_ [MPa]	-	-	-	-	-	0.0125	0.0225	0.041	-	0.01
Long Time Modulus G_∞_ [MPa]	-	-	-	-	0.0005	0.0025	0.0045	0.0078	-	0.002
Poisson’s Ratio ν [−]	0.42	0.23	0.25	-	-	-	-	-	-	-
Decay coefficient [s^−1^]					500,000	80	80	400	0.45	80

**Table 2:** Velocities of the players’ heads just before the collision, obtained from motion analyses.

Case No.	Injured Payer	Velocities											
		Striking Player						Struck Player					
		Translational [m/s]			Rotational [rad/s]			Translational [m/s]			Rotational [rad/s]		
		x	y	z	x	y	z	x	y	z	x	y	z
1	Struck	3.6	−1.3	−2.4	−0.68	1.6	−2.1	−4.2	1.5	0.08	0.35	−0.27	−2.0
2	Struck	−5.7	0.44	−0.50	−0.021	−0.01	−0.77	3.1	0.67	−1.4	−2.9	1.3	0.90
3	Striking	−1.1	−4.1	−0.80	0.27	−0.13	−3.9	2.0	1.2	0.24	−0.29	−0.05	−2.1
4	Struck	3.9	0.47	1.8	−1.0	2.4	−0.42	1.7	0.92	−1.9	−0.57	5.5	1.6
5	Striking	−2.5	−1.6	−0.12	−1.4	1.0	−8.3	4.2	0.02	−0.13	−0.09	−0.20	0.09

## Data Availability

The original contributions presented in the study are included in the article; further inquiries can be directed to the corresponding author.
